# Impact of Diminished Expression of circRNA on Multiple Sclerosis Pathomechanisms

**DOI:** 10.3389/fimmu.2022.875994

**Published:** 2022-06-03

**Authors:** Marcin P. Mycko, Anna E. Zurawska, Igor Selmaj, Krzysztof W. Selmaj

**Affiliations:** ^1^ Department of Neurology, University of Warmia and Mazury in Olsztyn, Olsztyn, Poland; ^2^ Center of Neurology, Lodz, Poland

**Keywords:** multiple sclerosis, circular RNA, non-coding RNA, biomarkers, RNA–RNA interactions

## Abstract

Circular RNA (circRNA) molecules represent a novel and unique class of endogenous non-coding RNAs controlling the expression and function of microRNA (miRNA) and post-transcriptional regulation. Recent studies implicated circRNA in the pathomechanism of multiple sclerosis (MS). Hybridization microarray was used to define the circRNA profile in the peripheral blood mononuclear cells (PBMCs) from 20 untreated patients with relapsing–remitting MS (RRMS: 10 in relapse, 10 in remission) and 10 healthy controls (HCs). We analyzed close to 14,000 individual circRNAs per sample. The discovery set data were validated using quantitative reverse transcription-polymerase chain reaction (qRT-PCR) with an independent cohort of 45 RRMS patients (18 in relapse, 27 in remission) and 27 HCs. Microarray analysis revealed 246 circRNAs differentially downregulated (*P* < 0.05) in RRMS patients versus HCs. We validated two circRNAs of the three showing the lowest levels of differential expression in the RRMS remission group versus the HC group: hsa_circRNA_101145 and hsa_circRNA_001896. Their expression was significantly decreased during remission in RRMS (*P* = 0.0000332, FC = 0.385 and *P* = 0.0455, FC = 0.591, respectively) and in patients showing a lower level of disability (hsa_circRNA_101145, *P* = 0.0695; hsa_circRNA_001896, *P* = 0.0008). Bioinformatic analysis revealed 10 miRNAs interacting with these circRNAs in a complementary manner and led to the discovery of three protein-coding mRNAs downregulated in patients with RRMS during remission. These transcripts have been previously implicated in oxidative stress, blood–brain barrier permeability, microglia function, and extracellular matrix molecules altering the microenvironment and inhibiting oligodendrocyte progenitor cells. circRNAs displayed a distinct profile in PBMCs from patients with RRMS, and our results may implicate circRNAs with low expression in important mechanistic pathways of RRMS.

## Introduction

Multiple sclerosis (MS) is a chronic inflammatory and demyelinating disorder of the central nervous system (CNS) (1, 2). While the etiology of MS is still not fully understood, accumulating evidence purports it to be an interplay between genetic, environmental, and epigenetic factors (3).

Results from genomic studies on MS did not lead to the discovery of strong genetic traits and rather suggested a complex of genes potentially involved in predisposition to the disease but with a modest odds ratio (4). More recently, several epigenetic mechanisms that might contribute to mechanisms in MS have been identified including microRNA (miRNA) alterations. These small RNA molecules are considered to be one of the most important post-transcriptional regulators, which might control the translation of several effector proteins (5). An increased miRNA expression downregulates protein synthesis and vice versa, and a diminished miRNA expression allows for enhanced protein generation. It was found that several miRNAs are implicated in the development of many disorders. Previous studies also showed miRNA involvement in MS and the induction of autoimmune reactions (6).

Circular RNA (circRNA) molecules represent a novel and unique class of endogenous non-coding RNAs controlling the expression and function of miRNA (7). Although circRNA might control transcription *via* several mechanisms like binding and sequestering RNA-binding proteins (RBPs) and base pairing with other miRNA types of transcripts, the major model of circRNA function depends on the miRNA sponge-type of activity (8). A single circRNA molecule can bind and neutralize several miRNAs simultaneously, and thus, might determine the availability of miRNA for post-transcriptional regulation. Thus, circRNAs might serve as a higher level of regulatory mechanism of miRNA (9). In addition, circRNAs are characterized by exceptionally high stability and tissue-specific expression making them excellent candidates for biomarkers among other types of RNA (10). Interestingly, accumulating evidence reveals circRNAs to have an active role in the CNS pathology and immune regulation (11). In our previous publication, we have found a number of circRNAs differentially expressed in immune cells of MS patients and a correlation between a group of overexpressed circRNAs and disease activity (12). Since circRNAs may demonstrate their regulatory potency over miRNAs either by increased or decreased expression, in the current study, we provide data on circRNAs with low expression and correlate them with disease stages and activity. We also evaluate the involvement of circRNAs with diminished expression in the decreased buffering of the regulatory activity of miRNAs, and consequently, their influence on the transcription of annotated protein genes. Our data support the important role of the circRNA–miRNA–mRNA regulatory network in the mechanisms of MS.

## Materials and Methods

### Subjects and Study Design

A total of 102 participants of Caucasian race were enrolled, comprising 65 patients with relapsing–remitting multiple sclerosis (RRMS) and 37 healthy controls (HCs) (**Table 1**). All patients with RRMS fulfilled the McDonald criteria (13). All patients had not been treated with disease-modifying drugs for at least 6 months. None had a history of anti-CD20, cladribine, and anti-CD52 treatment. The disability level for the neurological status of MS was assessed by the Expanded Disability Severity Scale (EDSS). Relapse was defined as the development of new neurological symptoms or worsening of existing symptoms leading to an EDSS increase by 1 point over at least 24 h. Patients in relapse were sampled within 3 days from the beginning of the relapse and before the administration of glucocorticoid treatment. Remission was defined as stable clinical condition for at least 6 months.

**Table 1:** Main characteristics of the individuals enrolled in the study.

Characteristic	Healthy controls	RRMS relapse	RRMS remission
Discovery set			
No.	10	10	10
Female/male/*P* versus controls	6/4/*P* = 1.00	6/4/*P* = 1.00	6/4/*P* = 1.00
Age, years, mean ± SD/*P* versus controls	35.8 ± 7.4/*P* = 1.00	35.2 ± 9.0/*P* = 0.93	32.4 ± 9.0/*P* = 0.80
Disease duration, mean ± SD	–	4.7 ± 6.0	5.0 ± 6.4
EDSS, mean ± SD	–	3.4 ± 1.9	2.4 ± 1.1
Validation set			
No.	27	18	27
Female/male/*P* versus controls	21/6/*P* = 0.85	11/7/*P* = 0.22	19/8/*P* = 0.53
Age, years, mean ± SD/*P* versus controls	34.7 ± 8.7/*P* = 1.00	36.8 ± 15.9/*P* = 0.85	36.3 ± 10.7/*P* = 0.85
Disease duration, mean ± SD	–	6.5 ± 9.2	5.0 ± 5.7
EDSS, mean ± SD	–	3.0 ± 0.8	1.5 ± 1.5

In the exploratory stage of our investigation, we performed microarray hybridization analysis of RNA from the peripheral blood mononuclear cells (PBMCs) of 20 patients with RRMS (10 in relapse, 10 in remission) and 10 HCs. In the second stage, for further validation, RNA samples were isolated from a new cohort of 45 patients with RRMS and 27 HCs and were subjected to quantitative reverse transcription-polymerase chain reaction (qRT-PCR) analysis. The clinical and demographic characteristics of the participants are presented in **Table 1**.

### MRI Protocol

Brain MRI data were acquired on a 3.0-T scanner (Siemens, Erlangen, Germany). MRI images used for this study were obtained ±2 weeks from the time of blood collection. To detect focal white matter lesions, MRI used dual-echo [repetition time (TR) = 4,500 ms, echo time (TE) = 22 and 90 ms, 25 slices, slice thickness = 3 mm, 512 × 512 × 44 matrix, field of view (FOV) = 250 mm] and T1-weighted images (TR = 750 ms, TE = 17 ms, 25 slices, slice thickness = 3 mm, FOV = 250 mm). Gadolinium (Gd)-enhanced T1-weighted lesions were identified from post-contrast T1-weighted spin-echo images (TR = 467 ms, TE = 8 ms, 240 × 240 × 132 mm FOV, number of excitations = 1), acquired 5 min after the administration of a dose of contrast (0.1 mM/kg).

### PBMC and RNA Isolation

PBMCs were isolated from whole blood by a density gradient centrifugation method using Ficoll Histopaque (Sigma-Aldrich, St. Louis, MO), as described earlier (14). Total RNA was extracted from PBMCs using the mirVana™ miRNA Isolation Kit (Life Technologies, Cleveland, OH). The RNA quantity and quality were measured with NanoDrop ND 1000 (Agilent Technologies, Santa Clara, CA).

### Microarray Hybridization and Quantitative RT-PCR Analysis

RNA samples were subjected to microarray hybridization with Arraystar’s Human Circular Array V2 (8 × 15K, Arraystar, Rockville, MD) as already described (12). Agilent Feature Extraction software (version 11.0.1.1) was used to analyze the acquired array images. Quantile normalization and subsequent data processing were performed using the R software limma package.

Quantitative validation of circRNA was performed by qRT-PCR with SYBR green using specific primers for circRNA identified in the discovery segment of our study. For qRT-PCR, cDNA was synthesized from total RNA using SuperScript III Reverse Transcriptase (Invitrogen) from 1 μg of the total RNA. Subsequently, the qRT-PCR reaction was performed in a total volume of 10 μl, containing 0.5 μl/10 μM of forward/reverse primers, 2 μl of cDNA, 2× SuperArray PCR master mix, and 2 μl of double-distilled water. The primers used in the qRT-PCR were designed such that the amplicon spanned the circular junction site as follows: for hsa_circRNA_101145, 5′ATGCTAAAACAAGATAAAGATGCCA3′ and 5′GGTGGGGAGAATAACTCATTTGG3′; for hsa_circRNA_001896, 5′TGTGCTCCGTGAGGATGAG3′ and 5′ATCTGAAAAATCCCTACGTGG3′; and for hsa_circRNA_100772, 5′ACAACCAAAGCAAAACGAT3′ and 5′GATCACGAATCTGTTCATTATATAC3′. β-Actin was used as an internal control using the following primers: 5′GTGGCCGAGGACTTTGATTG3′ and 5′CCTGTAACAACGCATCTCATATT3′. Briefly, the cycle parameters for the PCR reaction were 95°C for 10 min, followed by 40 amplification cycles of a denaturing step at the same temperature for 10 s and an annealing/extension step of 1 min at 60°C. A melting curve analysis was performed to confirm that the amplicons were unique and specific. Quantification for each sample was generated by Rotor-Gene Real-Time Analysis Software 6.0 (Qiagen, rmantown, MD). The relative amount of the target gene was determined by calculating the ratio between the target and housekeeping genes using the 2−ddCt formula.

### circRNA Bioinformatic Target Prediction

The target miRNAs for differentially expressed circRNAs were predicted with Arraystar’s miRNA target prediction software based on TargetScan (15) and miRanda (16) software tools including composite metrics allowing a search for the presence of circRNA sites that might match the seed region of miRNA. Based on the number and potential strength of the circRNA–miRNA interactions, the top 5 were reported and showed the highest expression of the predicted miRNA interacting with a given circRNA.

The classification of protein-coding mRNA putatively controlled by miRNA and identified by circRNA analysis was performed with the help of the miRNA target prediction database miRWalk (version 3.0; http://mirwalk.umm.uni-heidelberg.de/). miRWalk contains information that applies a random forest-based approach to integrate and predict miRNA target sites. The output of the random forest model is the predicted probability that a candidate target site is a true target site. Each of the miRNAs predicted to be affected by circRNAs is queried into the miRWalk to produce a list of candidate mRNAs with a predicted *P-*value <0.05.

### Statistical Analysis

Student’s *t*-test and chi-square test were used to assess the differences between the gender and age distribution of the patients enrolled in the discovery and validation sets. In the discovery set, candidate circRNAs were chosen if significant differences (*P* < 0.05) were observed among the groups using the one-way analysis of variance (ANOVA) test with the Scheffé *post-hoc* test. Prior to the ANOVA test, Levene’s test for equality of variances was performed. The *P*-values for each analysis of the discovery set were also adjusted for multiple comparisons using the approach of Benjamini and Hochberg to control for false discovery rate (FDR) at 0.05. In the qRT-PCR validation set, the association and the levels of each of the selected circRNAs between groups were assessed by the ANOVA test with the Scheffé *post-hoc* test, and the associated *P*-values and the area under the curve (AUC) from the receiver operating characteristic (ROC) for the full model were calculated. Outlier sensitivity analysis of the circRNA levels was tested using the Tukey method value (Tukey test). The power of the validation study was calculated according to the number of samples and determined as parameter alpha = 0.9. *R* ^2^ statistic was used to examine the association between circRNA expression and each of the clinical measures.

## Results

### Global Profiling of Downregulated circRNAs in the PBMCs of MS Patients

**Figure 1:**
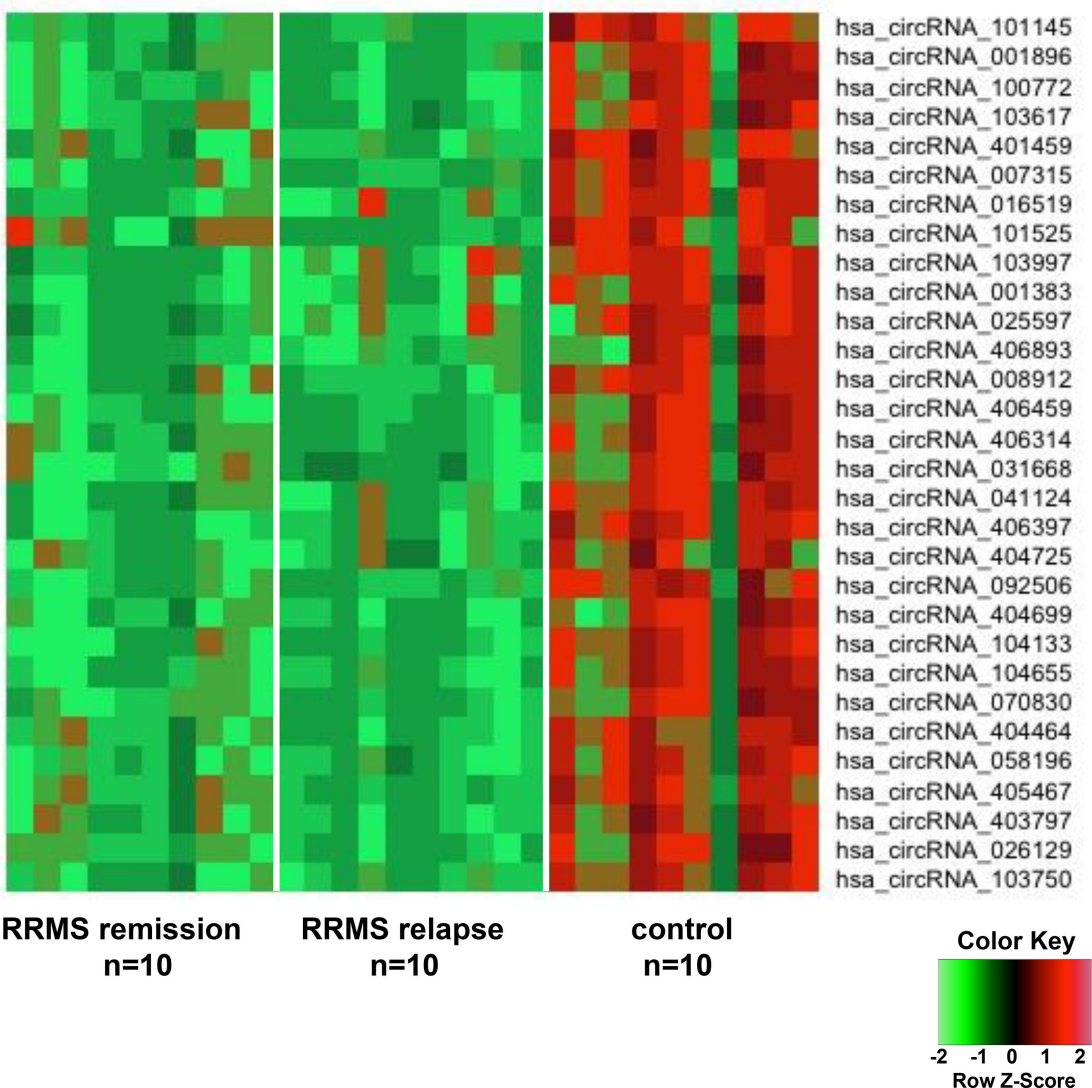
The heatmap shows differentially downregulated circular RNAs (circRNAs) from unsupervised hierarchical clustering present in the peripheral blood mononuclear cells (PBMCs) of patients with relapsing–remitting multiple sclerosis (RRMS) in the remission group versus the relapse group versus controls.

In order to discover the global expression profile of circRNAs from the PBMCs of patients with RRMS, we used microarrays containing probes for 13,617 human circRNAs (12). To search for unique circRNAs in the RRMS patients, we performed a global statistical analysis to detect differentially expressed transcripts. Apart from the differentially upregulated circRNAs that we analyzed in our previous publication (12), we also found 246 unique sequences that were downregulated in either RRMS relapse or remission when compared to HCs (*P* < 0.05, FC < 0.5). Unsupervised hierarchical clustering based on the most significantly downregulated circRNAs in the RRMS relapse versus the remission group versus controls is displayed in **Figure 1**. The list of the 10 most significant circRNAs downregulated in RRMS is presented in **Table 2**.

**Table 2:** List of the 10 selected circRNAs differentially downregulated in RRMS versus healthy controls.

circRNA^a^	circBase name	RRMS		
		*P*-value	FDR	Fold change versus controls
hsa_circRNA_101145	hsa_circRNA_0028198	0.0000060612	0.000468085	0.4627922654639374
hsa_circRNA_001896	hsa_circRNA_0001446	0.00001509935	0.00072142	0.4699256070170044
hsa_circRNA_100772	hsa_circRNA_0021535	0.00001538337	0.000727753	0.4859265939850062
hsa_circRNA_103617	hsa_circRNA_0069382	0.00002309324	0.000727753	0.4859265939850062
hsa_circRNA_007315	hsa_circRNA_0007315	0.00004630932	0.001258917	0.4234333969965446
hsa_circRNA_025597	hsa_circRNA_0025597	0.00004708777	0.005659019	0.4897750399570722
hsa_circRNA_016519	hsa_circRNA_016519	0.00004993413	0.001318583	0.4976906160036200
hsa_circRNA_101525	hsa_circRNA_0035381	0.00007092862	0.001529139	0.4355190524777811
hsa_circRNA_103997	hsa_circRNA_0074816	0.00008694402	0.00169458	0.4976314732399671
hsa_circRNA_001383	hsa_circRNA_0000622	0.00009371754	0.001769956	0.4978849102186546

a According to Arraystar.

### Validation of Differentially Downregulated circRNAs

**Figure 2:**
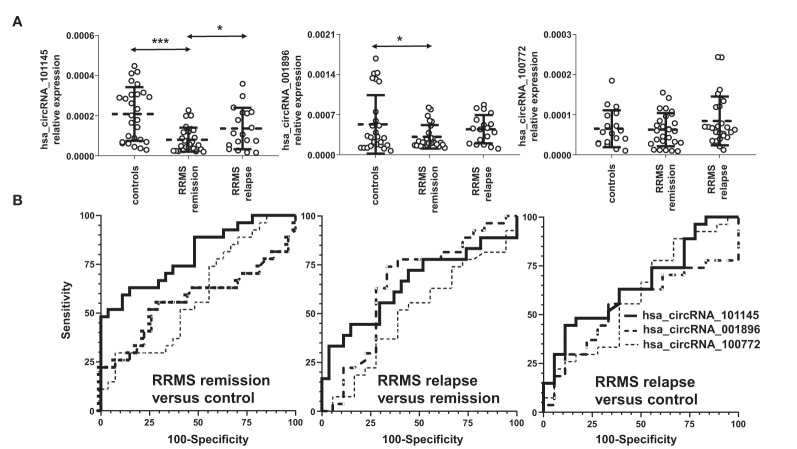
Validation of the differential downregulation of the selected circRNAs in RRMS. **(A)** The expression levels of the indicated circRNAs were analyzed *via* qRT-PCR. Significance was determined by analysis of variance (ANOVA) with the Scheffé *post-hoc* test. *P < 0.05, ***P < 0.0005. The dotted line represents the mean value ( ± SD). **(B)** Receiver operating characteristic (ROC) curves for the comparison of hsa_circ_101145, hsa_circ_001896, and hsa_circ_100772 expression levels in RRMS relapse versus control, RRMS remission versus control, and RRMS relapse versus remission.

To validate the differentially downregulated circRNAs, we tested the three circRNAs from the group of the highest differentially downregulated circRNAs in RRMS versus HC in the discovery cohort (**Table 2**). The validation cohort involved 72 subjects: in RRMS, 18 patients in relapse and 27 patients in remission and 27 HCs. The results showed that the expression levels of hsa_circRNA_101145 (*P* = 0.0000332, FC = 0.385) and hsa_circRNA_001896 (*P* = 0.0455, FC = 0.591) were significantly lower in RRMS patients in remission versus HCs. Furthermore, hsa_circRNA_101145 showed a significantly lower expression in RRMS remission versus relapse (*P* = 0.026, FC = 0.589). None of the tested three circRNAs showed significant differences between the relapse group versus the HC group, and the levels of hsa_circRNA_100772 were not significantly different between RRMS and HCs (**Figure 2A**).

To confirm further the significance of the observed differences in the expression of hsa_circRNA_101145 and hsa_circRNA_001896 between the RRMS groups and controls, we performed ROC analysis. Results confirmed that hsa_circRNA_101145 provided the best AUC value for discriminating RRMS remission patients from HCs (for hsa_circRNA_101145: 0.7984 ± 0.0596, *P* = 0.0002; for has_circRNA_001896: 0.5665 ± 0.0813, *P* = 0.4014) (**Figure 2B**). ROC analysis for hsa_circRNA_101145 also revealed better prediction for remission versus relapse (0.6523 ± 0.0892, *P* = 0.0864). In agreement with the analysis of expression validation, ROC analysis did not show a correlation between hsa_circRNA_100772 and MS remission or relapse. Collectively, the above data showed that the PBMC expression of hsa_circRNA_101145 provided the best distinction between RRMS patients in remission versus controls and RRMS patients in relapse.

### circRNA Downregulation Correlates With RRMS Clinical Severity

**Figure 3:**
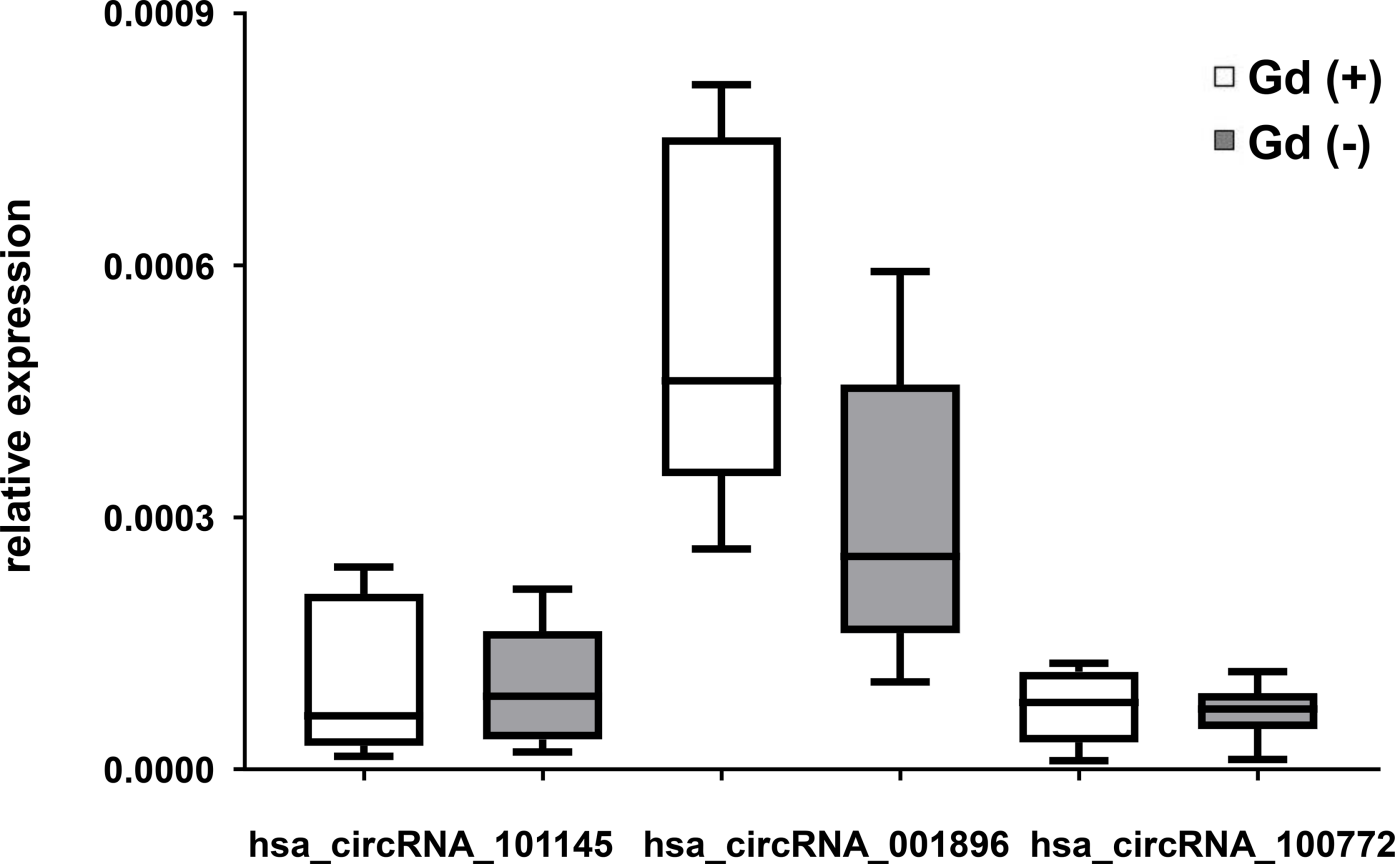
circRNA expression in PBMCs in relation to magnetic resonance imaging activity in RRMS. Box and whisker plots of the relative expression of the indicated circRNAs in PBMCs in gadolinium-enhanced (Gd)+ (*n* = 8) and in (Gd)− (*n* = 13) RRMS patients. Significance was determined by Student’s *t*-test.

To determine whether the expression of the circRNA hsa_circRNA_101145, hsa_circRNA_001896, and hsa_circRNA_100772 correlated with RRMS activity, we performed a correlative analysis between the level of circRNA expression and inflammatory activity measured by MRI. We divided the MS samples from patients in relapse into two categories, with gadolinium-enhancing lesions (Gd+; *n* = 8) and without (Gd−; *n* = 13) on MRI imaging. The results showed that patients with Gd− had a lower expression of hsa_circRNA_001896 than patients with enhancing lesions (Gd+); however, this difference was not statistically significant (*P* = 0.0671, FC = 0.658). The other two investigated circRNAs, hsa_circRNA_101145 and hsa_circRNA_100772, did not show a correlation with MRI activity (**Figure 3**). Thus, we confirmed that the lower expression of hsa_circRNA_101145, hsa_circRNA_001896, and hsa_circRNA_100772 was not associated with the MRI status of acute inflammatory activity.

**Figure 4:**
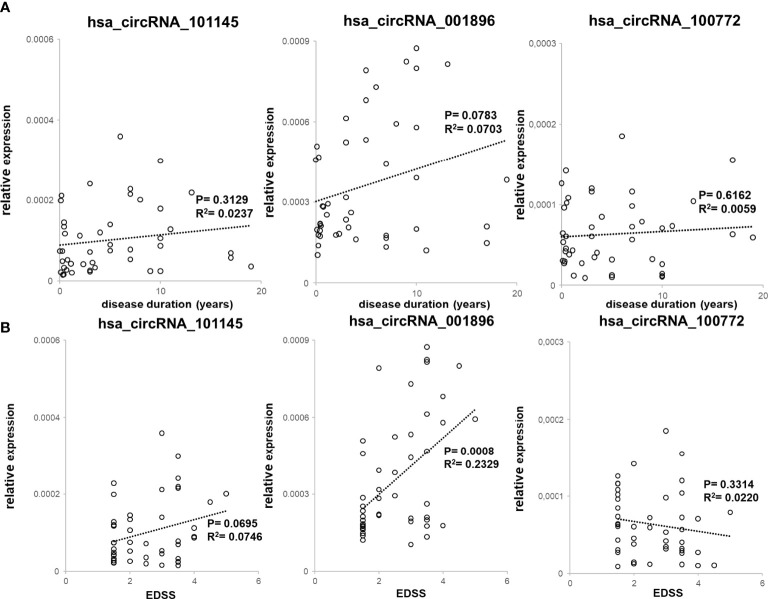
Correlation between downregulated circRNA levels with clinical RRMS parameters. **(A)** PBMC hsa_circRNA_101145, hsa_circRNA_001896, and hsa_circRNA_100772 expression levels were plotted against disease duration. Circles represent individual data, and the line represents a correlation trend; *P*-values were calculated by *R* ^2^ statistic. **(B)** PBMC hsa_circRNA_101145, hsa_circRNA_001896, and hsa_circRNA_100772 expression levels were plotted against EDSS. Circles represent individual data, and the line represents a correlation trend; *P*-values were calculated by *R* ^2^ statistics.

We also assessed the correlation between the levels of expression of two circRNAs (hsa_circRNA_101145 and hsa_circRNA_001896) found to be downregulated in MS remission, as well as hsa_circRNA_100772 expression, with disease duration (*n* = 45; **Figure 4A**) and with patient’s disability level, as measured by EDSS (*n* = 45; **Figure 4B**). All three circRNAs showed decreased levels in patients with a lower disability score (hsa_circRNA_001896, *P* = 0.0008; hsa_circRNA_101145, *P* = 0.0695; hsa_circRNA_100772, *P* = 0.6162). Thus, the correlative analysis of downregulated circRNAs in RRMS patients demonstrated a correlation with disability status, particularly for hsa_circRNA_001896. There was no correlation between downregulated circRNAs and disease duration.

### Identification of the Spectrum of Molecular Targets for hsa_circRNA_101145 and hsa_circRNA_001896

In order to investigate the potential functions of differentially downregulated circRNAs (hsa_circRNA_101145 and hsa_circRNA_001896) and their networking with miRNA, we annotated miRNA sequences that are known putative targets of these circRNAs. In **Table 3**, we present the data of the top 5 miRNAs for each of the two differentially expressed circRNAs defined by the presence of miRNA response elements.

**Table 3:** Validated differentially downregulated circRNAs in RRMS and the top 5 miRNA predicted to interact with them.

circRNA	Predicted interacting miRNA
hsa_circRNA_101145	hsa-miR-153-5p, hsa-miR-7-5p, hsa-miR-493-3p, hsa-miR-205-3p, hsa-miR-181c-5p
hsa_circRNA_001896	hsa-miR-329-5p, hsa-miR-26a-2-3p, hsa-miR-136-5p, hsa-miR-153-5p, hsa-miR-26a-1-3p

**Figure 5:**
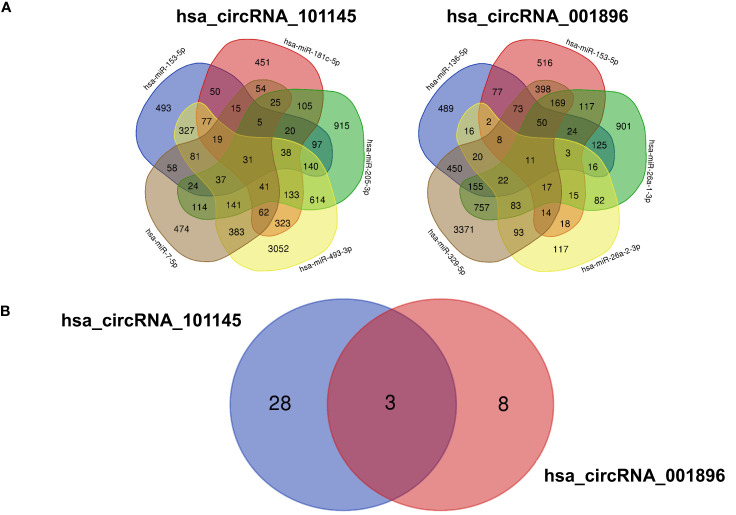
mRNA predicted from miRNA identified as interacting with circRNA. **(A)** The Venn diagrams display the overlap of the mRNA predicted from the hsa_circRNA_101145-regulated miRNA (hsa-miR-153-5p, hsa-miR-7-5p, hsa-miR-493-3p, hsa-miR-205-3p, hsa-miR-181c-5p) and hsa_circRNA_001896-regulated miRNA (hsa-miR-329-5p, hsa-miR-26a-2-3p, hsa-miR-136-5p, hsa-miR-153-5p, hsa-miR-26a-1-3p). The figures indicate the number of overlapping mRNAs regulated by the corresponding miRNAs. **(B)** The Venn diagram of the overlapping mRNA regulated by all miRNAs predicted to interact with hsa_circRNA_101145 and hsa_circRNA_001896. Figures indicate the number of overlapping mRNAs regulated by all miRNAs predicted from a single circRNA.

To further characterize the function of differentially downregulated circRNAs, we analyzed the potential impact of the circRNA–miRNA interactive complex on protein-coding transcripts. The miRNA target database search revealed a number of mRNAs that might be targeted by miRNAs controlled by circRNAs (hsa_circRNA_101145 and hsa_circRNA_001896) (**Figure 5A**). For hsa_circRNA_101145, there were 31 annotated mRNAs simultaneously controlled by all 5 miRNAs predicted to be interacting with this circRNA. For hsa_circRNA_001896, there were 11 annotated mRNAs simultaneously controlled by all 5 miRNAs predicted to be interacting with this circRNA. Subsequently, we analyzed the shared annotated mRNAs for both differentially downregulated circRNAs. We found that three mRNAs overlapped with both hsa_circRNA_101145 and hsa_circRNA_001896 (**Figure 5B**). These were mRNAs encoded by the genes dermatan sulfate epimerase (*DSE*), nitric oxide synthase 1 (*NOS1*), and NOP protein chaperone 1 (*NOPCHAP1*) (**Table 4**). Thus, the diminished expression of hsa_circRNA_101145 and hsa_circRNA_001896 might influence the post-transcriptional regulation of these three protein-coding genes.

**Table 4:** mRNA predicted to be reduced by hsa_circRNA_101145 and hsa_circRNA_001896 downregulation.

	Gene symbol	Gene name	Protein function
1.	*DSE*	Dermatan sulfate epimerase	Isomerase
2.	*NOS1*	Nitric oxide synthase 1	Oxidoreductase
3.	*NOPCHAP1*	NOP protein chaperone 1	Chaperone

Gene names and protein functions are defined according to the UniProt database (https://www.uniprot.org/).

## Discussion

A global analysis of the total RNA of the PBMCs of MS patients revealed several hundred circRNAs differentially expressed in MS versus HCs (12). A distinct pattern was identified between patients with RRMS relapse and remission and controls. Although the majority of differentially expressed circRNAs were upregulated, we also found a number of circRNAs that were downregulated in MS patients. Since circRNAs may demonstrate their miRNA-dependent regulation of post-transcriptional mechanisms also by decreased expression, in this study, we provide data on circRNAs with a low expression from the PBMCs of MS patients. The results from the global hybridization assay and subsequent PCR validation in a separate cohort of individuals identified two circRNAs with significantly diminished expression—hsa_circRNA_101145 and hsa_circRNA_001896—in MS patients in remission. We provided a prediction analysis on how these two circRNAs might influence miRNA programming and miRNA-dependent changes in protein transcripts. In addition, these two circRNAs showed a significant correlation with decreased disability, thus indicating their potential role in the mechanisms of disease progression.

circRNAs are a novel and unique class of endogenous RNAs, which are characterized by a covalently closed cyclic structure lacking polyadenylated tails (8). circRNAs have emerged as critical post-transcriptional regulators of gene expression by binding to miRNA and buffering their repression of mRNA targets (7). The regulatory property of circRNAs over miRNAs has been implicated in several important biological processes, such as cell differentiation and immune cell polarization, and several other immune processes (17). The above activity directly links circRNAs to the regulation of immune reactions and immune-mediated disorders. In addition, the abundance of circRNAs in the CNS suggests that they might have particular relevance to the pathologic processes in nervous system diseases (11).

Thus, this study has resulted in the identification of two circRNAs and a group of 10 miRNAs that might be targeted by circRNAs in RRMS patients in remission, which differentiate them from HCs. A diminished expression of circRNA would allow for an enhanced activity of all these 10 miRNAs. Several miRNAs have been linked to autoimmune processes leading to demyelination in MS (18). We and others have previously identified miRNA connected to the development and maintenance of encephalitogenic populations of T helper cells (19–21). Indeed, reports on miRNAs in MS patients have shown them to be differentially expressed in PBMCs (22), whole blood, T cells, and B cells (19, 23). Within the group of miRNAs identified in this study, the increased presence of miR-181c in the cerebrospinal fluid has been linked with a higher risk for clinically isolated syndrome (CIS) conversion to confirmed MS (24). Although the other miRNAs have not been directly linked with MS, almost all of them have been found to be involved in processes that might operate in autoimmunity and brain recovery and MS-related processes like autophagy, apoptosis, neural stem cell proliferation, and neurodegeneration (25–28).

The data obtained herein lend a strong MS-related context to the action of the two circRNAs highlighted. Accordingly, circRNAs have already been implicated in autoimmune pathology including MS. It was reported that the circRNA hsa_circ_0106803, derived from the *GSDMB* gene, was 2.8-fold upregulated in RRMS patients (29). More recently, other groups have reported on RNA-seq profiling of leucocytes and showed sex-dependent circRNA upregulation in MS patients (30, 31). Bioinformatic analysis of circRNAs of non-coding elements in the MS-associated genome suggested that they may be possibly involved in susceptibility to the disease (32). In another study, exosomal circRNA within the cerebrospinal fluid was found to correlate with IgG levels in MS (33). We have also recently reported on differentially overexpressed circRNAs in MS patients that modulate B-cell function (12). Of interest, all these studies reported on the increased expression of circRNAs leading to decreased miRNA regulation of post-transcriptional processes. This study provides for the first time data on the diminished expression of circRNAs in immune cells of MS patients, and thus, might identify the miRNA-dependent diminished regulatory mechanism of protein-coding transcripts related to MS.

It is of particular relevance that we have defined by *in-silico* analysis three protein-coding genes controlled by both circRNA molecules identified in this study: *NOS1*, *NOPCHAP1*, and *DSE*. *NOS1* was repeatedly implicated in oxidative stress in immune cells and tissue in MS, blood–brain barrier permeability, and microglia function (34, 35). Dermatan sulfate, chondroitin sulfate, and heparan sulfate are covalently attached to specific core proteins to form proteoglycans in their biosynthetic pathways. It is of particular interest that chondroitin sulfate can heavily influence tissue remodeling and inhibit remyelination in MS (36). Both chondroitin sulfate and other extracellular matrix molecules deposited into lesions provide an altered microenvironment that inhibits oligodendrocyte progenitor cells. Recently, a new potential therapeutic approach has been initiated to target extracellular matrix components to reduce detrimental neuroinflammation and to promote recruitment and maturation of oligodendrocyte lineage cells to enhance remyelination (37).

Another aspect of our findings might prompt consideration of the enhanced expression of miRNAs resulting from diminished circRNA expression as potential distinguishing biomarkers for MS. The complexity of the diagnostic MS procedure makes it necessary to search for simpler methods that would permit and enable rapid and reliable diagnosis. In this regard, a particularly important role can be played by miRNA. The two differently downregulated circRNAs shown here had a group of miRNAs targeted by them. As a result of diminished circRNA expression, all these miRNAs operate at an increased level. These single-stranded non-coding RNA molecules of 21–23 nucleotides in length that regulate the expression of genes encoding proteins unexpectedly showed a remarkable stability in body fluids (22). In previous studies, miRNAs have been proven to serve as biomarkers of various pathological and clinical conditions. Several studies reported increased miRNA expression in MS patients (14, 38, 39). As indicated above, miR-181c, one of the miRNAs highlighted in this study, has already been correlated with a higher risk of CIS conversion to MS (24). In addition, miR-181c distinguishes the relapsing–remitting form of MS from primary progressive (40). miRNA expression also correlated with neurodegenerative processes in MS influencing disability (41), as observed in this study. Thus, it is anticipated that further studies on the circRNA–miRNA circuit might facilitate the delineation of novel diagnostic and prognostic biomarkers in MS.

In summary, we have demonstrated the downregulation of the circRNA molecules hsa_circRNA_101145 and hsa_circRNA_001896 in the PBMCs of patients with RRMS in remission. The expression pattern of circRNAs that interact with miRNA regulatory mechanisms creates a circRNA–miRNA network specifically influencing post-transcriptional regulation. The alteration in this network might provide microenvironmental changes modulating MS progression. These data hold promise that further studies on circRNAs might progress our understanding of the pathological processes of MS as well as contribute to the search for MS biomarkers.

## Data Availability Statement

The datasets presented in this study can be found in online repositories. The names of the repository/repositories and accession number(s) can be found below: https://www.ncbi.nlm.nih.gov/geo/, GSE171950.

## Ethics Statement

The studies involving human participants were reviewed and approved by the Ethics Committee of the University of Warmia and Mazury in Olsztyn, Poland. The patients/participants provided their written informed consent to participate in this study.

## Author Contributions

MM was involved in the study concept and design, data acquisition and analysis, and data interpretation; MM also drafted the contents of the article. AZ had a major role in the data acquisition and analysis as well as data interpretation. IS had a major role in the data acquisition and analysis. KS was involved in the study concept and design, data acquisition and analysis, and data interpretation; KS was also involved in drafting the contents of the article. All authors contributed to the article and approved the submitted version.

## Funding

This study was supported by the National Science Centre Poland OPUS 2016/23/B/NZ6/02541 to MM and by the University of Warmia and Mazury in Olsztyn internal grants to KS, MM, and AZ.

## Conflict of Interest

The authors declare that the research was conducted in the absence of any commercial or financial relationships that could be construed as a potential conflict of interest.

## Publisher’s Note

All claims expressed in this article are solely those of the authors and do not necessarily represent those of their affiliated organizations, or those of the publisher, the editors and the reviewers. Any product that may be evaluated in this article, or claim that may be made by its manufacturer, is not guaranteed or endorsed by the publisher.
